# 1387. Prevalence and Risk Factors for Bacterial Resistance in Diabetic Foot Infections at a Hospital in Nicaragua.

**DOI:** 10.1093/ofid/ofac492.1216

**Published:** 2022-12-15

**Authors:** Kevin A Sandoval-Rojas, Roger Maliaños-Miranda, Sunaya Marenco-Avilés, Maikelyn A Castillo-Cano, Guillermo D Porras-Cortés

**Affiliations:** Hospital Dr. Fernando Vélez Paiz, Managua, Managua, Nicaragua; Hospital Dr. Fernando Vélez Paiz, Managua, Managua, Nicaragua; Hospital Dr. Fernando Vélez Paiz, Managua, Managua, Nicaragua; Hospital Dr. Fernando Vélez Paiz, Managua, Managua, Nicaragua; Hospital Dr. Fernando Vélez Paiz, Managua, Managua, Nicaragua

## Abstract

**Background:**

The bacterial resistance could have an deleterious outcome in patients with diabetic foot infections (DFI). The aim of this study was to determine the prevalence, the main risk factors, and clinical outcomes for DFI caused by bacterias that were resistant to different classes of antimicrobials in a hospital in Nicaragua.

**Methods:**

A case-control study was conducted between January 2019 and March 2020 at Hospital Dr. Fernando Vélez Paiz in Managua (Nicaragua). Cases were patients with diabetic foot infections caused by drug-resistant organisms (DRO) and the controls were patients infected by non-drug resistant organisms (NDRO). The resistance to quinolones, beta-lactams, and carbapenems were evaluated in the Gram negative bacterias. In the cases of *Staphylococcus aureus* the resistant to methicillin was evaluated. Samples of tissue and exudates obtained by aspiration were processed. Conventional methods as blood agar and McConkey agar were used, and the final identification method was Vytek 2 System. Different variables of both groups were compared.

**Results:**

A total of 76 bacterias were identified in 61 patients. Forty-four patients had an infection with at least one resistant bacteria for a prevalence of 72%. *Staphylococcus aureus* was the most prevalent bacteria and 76.9% of them were resistant to methicillin (MRSA). A 71% of the Gram negative bacterias were resistant to at least one class of antibiotic; 58% of them were resistant to quinolones, 40% were ESBL positive, and 12% were resistant to carbapenems. A total of 12 isolates were multi-drug resistant organisms (MDRO). The main risk factor to have an infection with a resistant bacteria was the use of antibiotics into the previous 3 months (OR:4.28; CI95%: 1.31-13.98). An infection caused by DRO was associated with a increased risk of amputation. Infections caused by NDRO had more probability of clinical resolution (OR:0.14; CI95%:0.04-0.48).

Prevalence of Resistant Bacterias to Different Classes of Antibiotics in Diabetic Foot Infections.

**Figure:**
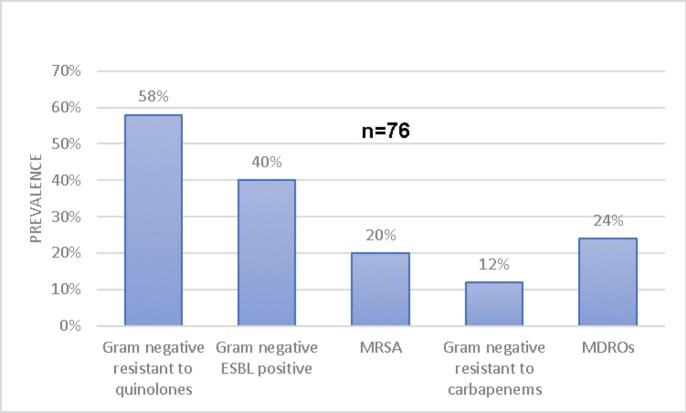

**Conclusion:**

In this setting, the bacterial resistance in diabetic foot infections has to be considered significative. MRSA had a high prevalence and the Gram negative organisms showed an important resistance rate to different classes of antibiotics. The main risk factor was the previous use of antibiotics. The clinical outcome is affected by the bacterial resistance.

**Disclosures:**

**All Authors**: No reported disclosures.

